# Big Data Analytics in the Fight against Major Public Health Incidents (Including COVID-19): A Conceptual Framework

**DOI:** 10.3390/ijerph17176161

**Published:** 2020-08-25

**Authors:** Qiong Jia, Yue Guo, Guanlin Wang, Stuart J. Barnes

**Affiliations:** 1 Department of Management, Hohai Business School, Hohai University, Nanjing 211100, China; jiaqionghit@163.com (Q.J.); kalynne98@163.com (G.W.); 2 The Department of Information System and Management Engineering, Faculty of Business, Southern University of Science and Technology, 1088 Xueyuan Avenue, Shenzhen 518055, China; guoy@sustech.edu.cn; 3 CODA Research Centre, King’s Business School, King’s College London, Bush House, 30 Aldwych, London WC2B 4BG, UK

**Keywords:** COVID-19, big data analysis, major public health incidents, epidemic prevention and control, visual analysis, deep learning, predictive analysis

## Abstract

Major public health incidents such as COVID-19 typically have characteristics of being sudden, uncertain, and hazardous. If a government can effectively accumulate big data from various sources and use appropriate analytical methods, it may quickly respond to achieve optimal public health decisions, thereby ameliorating negative impacts from a public health incident and more quickly restoring normality. Although there are many reports and studies examining how to use big data for epidemic prevention, there is still a lack of an effective review and framework of the application of big data in the fight against major public health incidents such as COVID-19, which would be a helpful reference for governments. This paper provides clear information on the characteristics of COVID-19, as well as key big data resources, big data for the visualization of pandemic prevention and control, close contact screening, online public opinion monitoring, virus host analysis, and pandemic forecast evaluation. A framework is provided as a multidimensional reference for the effective use of big data analytics technology to prevent and control epidemics (or pandemics). The challenges and suggestions with respect to applying big data for fighting COVID-19 are also discussed.

## 1. Introduction

In recent years, with the rapid development of economies and transportation facilities (such as air and high-speed rail), population mobility has increased, and once a major public health event occurs, it may cause significant losses to national economies and people’s lives. The severe acute respiratory syndrome (SARS) that occurred in 2003 and the new type of corona pneumonia virus (COVID-19 or SARS-CoV-2) is an example of major public health incidents. Taking the SARS outbreak in 2003 as an example, China’s GDP growth fell by 3% in the second quarter, foreign direct investment fell by $7.12 billion, and the tourism and catering industry decreased by $5 billion (international tourists) and $3.5 billion (domestic tourists) [1]. The spread of the new pandemic has led to a longer period of cessation of global economic activity, a greater demand shock, and these have spread to Europe, the United States, and other major economies. The cost of the virus in the world economy may reach $4.1 trillion [2]. The new coronavirus may cause global GDP to drop by nearly 1% in 2020 [3].

There are multiple factors that have caused these major economic losses. On one hand, due to the suddenness and novelty of COVID-19, there was a serious lack of knowledge about the highly infectious new virus, and it is difficult to quickly develop a safe vaccine. On the other hand, governments have not taken timely preventive measures to suppress the spread of the pandemic, resulting in severe damage to human health and societal stability. In addition, before and after the outbreak, because of the lack of an effective early warning, rapid response mechanisms, implementation of effective prevention and control decisions, the best prevention time have been missed. In order to respond efficiently and propose a preventative control plan, not only do we need a complete emergency management system, but also scientific data analysis for decision support. In the early stage of the outbreak, due to the limited data accumulated, the role of big data analysis was limited [4]. However, with the spread of the pandemic, the analysis results based on the gradually generated large amounts of data have played an important role in the tracking of people’s movements, early warning of high-risk areas, screening of asymptomatic potential infections, drug development, information release, and policy support. They have also become an important basis for the implementation of preventive control programs and have played an important role in enhancing the modernization level of national governance, promoting protection and improving people’s livelihood. Although many studies on COVID-19 have recently emerged, and various data science applications combating the pandemic have been reported recently [4], these studies have focused more on data sources and applications in a broad fashion without focusing on detailed guidelines for prevention and control to assist decision makers. Moreover, there is still a lack of a theoretical framework for big data analytics in the prevention and control of Major Public Health Incidents (including COVID-19). Thus, it is necessary to propose such a framework to focus on the prevention and control of COVID-19 using big data, which is also applicative in instances of other epidemic diseases. The proposed definitions, characteristics, data sources, applications, and framework can also enlighten and support the decisions of governments, enterprise, medical institutions, users, and researchers.

Therefore, based on enlightening experiences of big data application for fighting the pandemic, our study focuses on the theme of major public health incident prevention decision-making and aims to clarify the following research questions:

What are the characteristics of major public health incidents such as the COVID-19 pandemic and what difficulties does the international community face in preventing major health incidents?

What are the sources of big data that support the fight against major health incidents such as COVID-19?

In the stage when major public emergencies such as the COVID-19 pandemic are about to erupt or have already erupted, what decision-making framework can big data analysis technology provide?

What challenges and solutions can be concerned for applying big data analytics for fighting against COVID-19?

The first question will help us unravel and understand the attributes, characteristics, and difficulties of major public health incidents such as COVID-19. The second question helps us understand and summarize what massive data sources are currently available for government decision-making reference. The third question is how to use these big data to analyze the real problems after these data are available, and what results can be drawn for decision-making, so as to provide ideas for the government to quickly establish a preventive decision-making governance process. The last question is to discuss the challenges and solutions for utilizing the big data to fight against COVID-19.

## 2. Definition, Characteristics, and Prevention Difficulties of COVID-19

Public health incidents refer to sudden outbreaks of major infectious diseases, mass unexplained diseases, major food and occupational poisoning, and other events that have caused or may cause serious damage to public health. The World Health Organization (WHO) defines “unexpected public health incidents of international concern” as unusual public health risks to other countries through the international spread of disease that may require coordinated international event response. The WHO warning is divided into six major levels, from level one to level six, with level six being the highest level, indicating that the epidemic is spreading globally (a pandemic). Following this, each country also has their own policies; for example, China divides natural disasters, accidental disasters, and public health incidents into particularly important (level I), significant (level II), large (level III), and general (level IV) according to factors such as the degree of social harm and the scope of influence.

The outbreak of major public health incidents usually has the characteristics of suddenness, uncertainty, unpredictability, high hazard, high social concern, chain reaction, timely disposition, and evasive prevention [5]. The explanations of these features of COVID-19 are shown in Table 1.

Outbreaks of viruses such as COVID-19 are arduous to prevent. A key feature is in fact that it is an unknown, new type of coronavirus. People have very limited previous knowledge that is relevant. It also has high uncertainty and unpredictability. Since the virus is extremely contagious, with a high degree of suddenness and a strong chain reaction, it has caused a high degree of social attention due to its rapid spread around the world. Although its rate of mortality is about 5.6% lower than SARS, its spreading ability (diagnosis rate of close contacts) is very high, resulting in a high number of infections. Therefore, it is incumbent upon governments to deal with it in a timely fashion to avoid spread on a larger scale [10,11,12]. In addition, although transportation brings convenience to citizens, in the case of limited cognition and a lack of effective control measures, it can also accelerate the spread of the virus and infect large numbers of people with high death incidence in a short period of time. For example, from 20 January 2020 to 8 March 2020, 80,735 accumulated confirmed cases were reported in China, 58,600 cases were cured, 3119 persons had died from COVID-19, and 674,760 close contacts were tracked. The number of close contacts observed was 20,146 [13].

**Figure 1:**
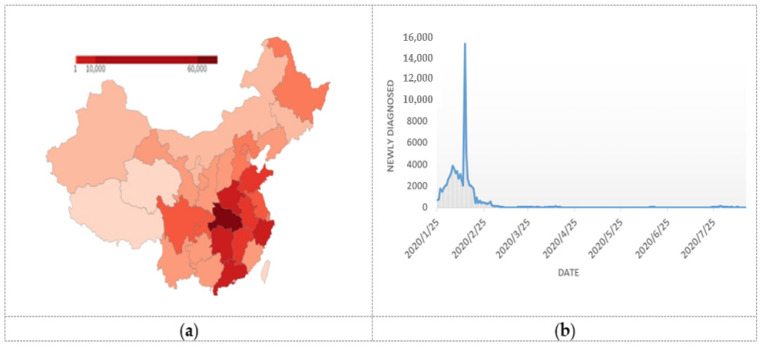
(**a**) Cumulative confirmed cases of COVID-19 pandemic in China [13]; (**b**) The development trend of COVID-19 pandemic in China: New diagnosed cases [13].

**Figure 2:**
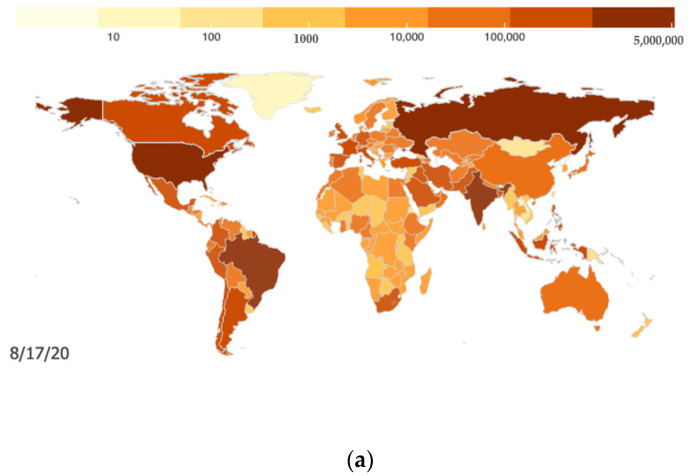

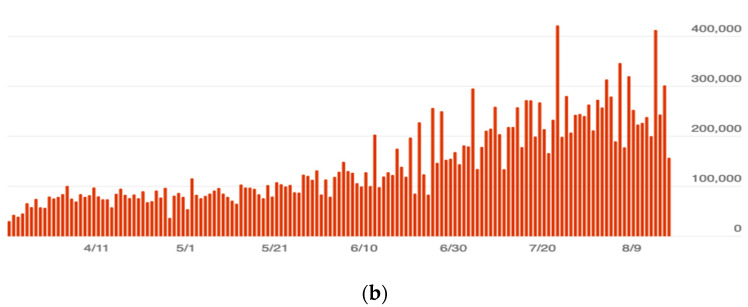
Global confirmed cases of the COVID-19 pandemic [14]. (**a**) COVID-19 pandemic across the globe; (**b**) Global daily new cases of the COVID-19 [14].

Faced with the emergency situation, the Chinese government responded quickly and enforced strict controls and timely resource deployment to minimize the loss and gradually control the epidemic. As shown in Figure 1, as of July 2020, under the policy intervention of the Chinese government with big data applications, the number of newly confirmed cases continues to decrease, while in other parts of the world, the confirmed cases do not yet appear to be controlled effectively, as shown by the number of confirmed cases in Figure 2. Overall, the global pandemic situation is not yet optimistic. By 18 August, 21,826,342 accumulated confirmed cases were reported in the world, 13,888,301 cases were cured, and 773,152 persons had died from COVID-19 [13]. Fighting against COVID-19 has become a long-term topic for the whole world, which is also transforming the situations of economies, politics, and societies.

## 3. Big Data Resources Associated with Major Public Health Incidents Such as COVID-19

**Figure 3:**
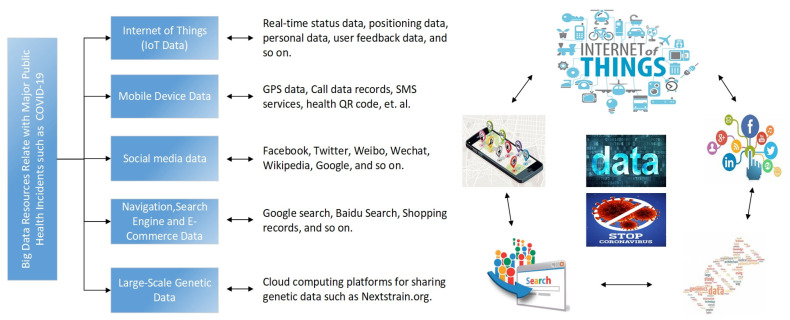
A taxonomy of big data resources associated with major public health incidents such as COVID-19.

By designing a new generation of technology and architecture, big data analytics can obtain high velocity, discover and analyze high volumes and varieties of data to extract value [15,16,17]. Characterized by the “4Vs”, with the rapid development of information technologies such as the Internet of Things (IoT), cloud computing and social networks, big data technology has been widely used in healthcare, transportation, finance, logistics and supply chains, manufacturing, and other industries [17]. Big data analytics have become an important technical support for the promotion of national modern governance. It is suggested by IBM that 90% of all global data was produced in the last two years [18]. Every Internet user generates on average 1.7Mb of data per second, and the data generated every day around the world is approximately 2.5 × 1018 bytes. The market for big data analysis is expected to reach $103 billion by 2023 [19]. With the big data platform, we can store big data in a distributed file system, perform big data batch calculation on historical data, and deploy big data stream processing calculation on data generated in real time. We can perform data analysis, data mining, and machine learning on the processed data. These new data storage and analysis technologies provide key management decision supports for people to analyze massive amounts of data. In terms of fighting against major public health incidents in the COVID-19 category, we have summarized the following big data sources that can be used to support and carry out pandemic prevention and control, as shown in Figure 3.

### 3.1. Internet of Things (IoT) Data

The Internet of Things is a network that connects any goods to the Internet through radio frequency identification (RFID), infrared sensors, global positioning systems, laser scanners, and other information sensing devices. By exchanging information and communicating, IoT can be used for the intelligent identification, positioning, tracking, monitoring, and management of goods [20]. Sensors can transmit a large amount of static and dynamic data in real-time [21]. These data include real-time status data, positioning data, personal data, and user feedback data. In the medical environment of COVID-19, real-time status data regarding patients, medical staff, and hospitals will generate a large amount of real-time data. In terms of patients, it mainly includes face, fingerprint, iris, genetic and other characteristic data; hospital, community, or remote visitor data; and patient portable medical equipment or wearable device data. During the treatment of infected patients, medical staff will also generate their own status and treatment-related feedback data in real time. For example, during the COVID-19 pandemic, medical personnel face a huge risk of infection. Through the IoT technology, built-in RFID chips in medical staff’s clothes, a clinicians’ dirty clothes management system, a medical staff health management system combining RFID tags and face recognition can send medical staff status data to a database in real time. In addition, in the community, to identify disease cases of COVID-19, IoT can also collect facial recognition data and conduct accurate infrared thermal imaging screening to check if people wear masks or not and body temperature so as to provide early warning [22]. The positioning data mainly come from the medical equipment and consumables in the hospital, the management of medical rescue materials, medical waste traceability, and so on, which is transmitted through RFID and analyzed for early warning [23]. For example, personalized behavior and user feedback data come from the contactless retail data of unmanned supermarkets supported by the IoT technology, which also provides a data source for analyzing consumer habits during the COVID-19 pandemic.

### 3.2. Mobile Device Data

Mobile device data mainly refer to data generated through mobile phones. The popularity of mobile devices means that it is now possible to track people’s movements and better understand the path of infectious diseases following privacy regulations. In the work of blocking the spread of the new coronavirus, there are three situations that make prevention and control difficult. First, it is difficult to know whether a person has contacted people who have been diagnosed in public transportation and other places where the virus originated. Secondly, individuals may have recently visited a pandemic site or contacted confirmed individuals, but they did not care, report, or actively segregate themselves. Thirdly, individuals may deliberately conceal their history of being in a pandemic area or contacting the diagnosed individuals. These problems have severely affected the effectiveness of pandemic prevention. However, without prejudice to personal privacy, GPS coordinates obtained from mobile phone data enable experts to track whether people have been in contact with infected cases. This helps identify and isolate these infected people and treat them in advance. The mobile data of the infected population not only helps to understand the overall situation during the outbreak, but it also helps us predict how the disease will spread in future outbreaks and understand which interventions are the most effective [24]. For example, Thompson et al. [25] used call data records (CDRs) of mobile phones to study the impact of travel on the dynamics of malaria and rubella in Kenya, and they found that large-scale temporary fluctuations in mobile phone roaming have a significant correlation with related disease fluctuations. However, the mobile data from phones may not be able to represent all travel modes, so it is necessary to combine the analysis of data in a specific region of a country, such as a remote area, or to examine the macroeconomic level to reduce deviation. The application of mobile phones can also have a positive effect on the prevention and control of diseases. Velthoven et al. [26] reviewed a total of 21 related studies, and the scope, effectiveness, acceptability, and feasibility of the impact of mobile phone SMS services on HIV infection prevention, treatment, and care were evaluated. The researchers found that there are some (limited) evidences on the effect of mobile phone text messages on HIV care, and it is necessary to fully record the results and constraints of such projects in order to assess the impact of mobile phone messages on HIV disease care and obtain best practice methods. Sareen et al. [27] integrated data from mobile communications and IoT sensors to help government agencies prevent and control the Zika virus.

### 3.3. Social Media Data

The development of social media platforms provides a new stream of big data to carry out disease prevention and control [28]. Social media platforms such as Facebook, Twitter, Weibo, and WeChat have become indispensable daily life tools for people. Taking WeChat as an example, there are more than 1.15 billion active users every month [29]. At present, WeChat has opened the “pandemic inspection” function to provide prevention and control clues for the COVID-19 pandemic. By analyzing real-time data accumulated on these social platforms, we can gain a deeper understanding of the time and geographical location of disease transmission. An effective practice in China to fight against COVID-19 is the Quick Response (QR) code in healthcare, which is a two-dimensional barcode revealing the information of people’s travel records and health status. If the color of the code is green, people are free to proceed; otherwise, they are barred from entry. This application can be integrated into the WeChat or Alipay for ease of use [28]. When seasonal flu broke out in the United States, researchers used data on Twitter to predict better when the flu season will peak [30], significantly improving flu predictions [31]. Their research found that the prediction model based on Twitter data can reduce the error by 17–30% compared to the prediction model using only historical data. For a given level of accuracy, the predictions generated using Twitter data are 2–4 weeks ahead of the benchmark model. Salathé and Khandelwal [32] performed a semantic analysis of social platform data including the degree of fear during the outbreak. It shows that the data provided by the social media platform can not only help us to predict the development of the pandemic more accurately, but also estimate the psychological state of the public to effectively carry out media publicity.

McIver and Brownstein [33] counted the number of daily visits to certain Wikipedia articles related to influenza or health from December 2007 to August 2013. These data were compared with influenza-like illness (ILI) records provided by the Centers for Disease Control and Prevention to accurately estimate the level of ILI activity in the US population. Based on a combination of Wikipedia article access log data and influenza disease reports, Hickmann et al. [34] created weekly forecasts of seasonal influenza. For large-scale text datasets of media reports and literature, the combination of machine learning, natural language processing, and experts can effectively predict disease outbreaks [35]. In the more than two months since the outbreak of COVID-19, public reports and academic research literature databases on the Internet have also increased rapidly, providing details of familiar features of the disease and rich text data for further research. As of August 16, 2020, there have been more than 1,270,000 reports and research papers in Google Scholar increasing from about 688 in March, and more than 41,888 articles published by Web of Science, increasing from about 266 in March. The analysis of these research texts has scientific significance for guiding the prevention and control of the pandemic.

### 3.4. Navigation, Search Engine, and E-Commerce Data

Similar to social media data, navigation and search engine data are an important source of disease prevention and control big data. They are not directly related to medical treatment or disease diagnosis, but their potential information can reflect the development of the disease and can capture people’s attention toward some diseases. Google released the Google Flu Trends (GFT) flu forecast product as early as the year 2008. It analyzed the users’ search term records to predict the flu pandemic. Although the H1N1 outbreak was successfully predicted in 2009 [36], it was also questioned because of the high predicted incidence of influenza [37]. However, with the change of search engine strategy, combining with the traditional control dataset, considering the causality of the problem rather than paying too much attention to the statistical characteristics of the data, big data analysis results based on search data may still have reference value.

**Figure 4:**
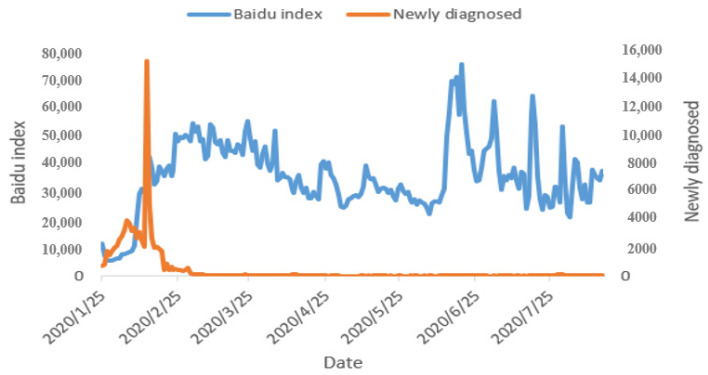
COVID-19’s Baidu index (blue) and the number of newly diagnosed cases (orange).

Based on search engine big data, decision-makers can capture user needs and hotspots in real time to assist in pandemic prevention decisions. For example, when implementing isolation policies, the Baidu index of hot search topics changes with concerns and needs, providing real-time adjustments to pandemic prevention policies. For example, the Google search volume for “COVID-19” as a keyword soared from 1 March 2020 to reach a peak on 28 March 2020 [8]. As shown in Figure 4, the trend of Baidu index (blue) and newly confirmed COVID-19 cases (orange) show a certain correlation, demonstrating the potential relationship between public opinion and pandemic.

For example, new diagnoses reached the highest peak in China of 15,151 on 12 February 2020, which subsequently triggered the local peak of the Baidu index. In addition, “84 disinfectant”, “how to use a thermometer”, “N95 mask”, “reagent kit”, and so on, displayed on the Internet hot search have become the focus of material deployment, publicity, and education. In addition to searching data, most people can only shop online at home, and this generates a large amount of e-commerce data. These data provide references for pandemic prevention and control by understanding user needs during the pandemic, material allocation, product innovation after the pandemic, and industry development.

### 3.5. Large-Scale Genetic Data

**Figure 5:**
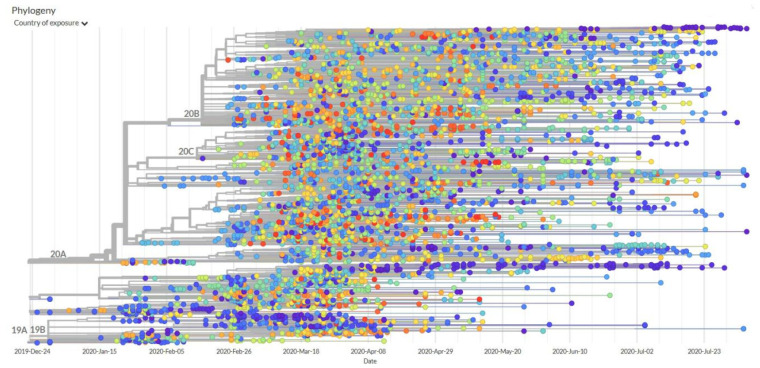
Nextstrain platform analysis of new coronavirus transmission genome [39].

Efficient genome sequencing methods can accumulate large amounts of data to help the public conduct in-depth analysis of mutant micro-organisms in real time [38]. The genetic data from pathogens play an important role in finding the source of the virus, drug and vaccine development, and clinical diagnosis. Notwithstanding, there may be a variety of reasons, such as delays in sample collection and testing, the lack of collaboration and reporting tools, or the deliberate preservation of first-hand data for the purpose of pre-posting articles, all of which can become obstacles to obtaining genetic data that hinder research and development. In order to solve this problem, scientists and medical institutions in various countries are currently building various development source cloud computing platforms, such as Nextstrain.org [39], which allow researchers to share and track genome sequences in real time, providing a reliable and real-time source for analyzing diseases. The large-scale genetic data of the global research community is collected on the platform, and the phylogenetic tree of virus transmission can be visualized. Figure 5 shows a genomic analysis of the evolution of the new coronavirus generated by the platform.

This phylogeny shows evolutionary relationships of hCoV-19 viruses from the ongoing novel coronavirus COVID-19 pandemic. This phylogeny reveals the emergence of COVID-19 in November to December 2019, followed by sustained human-to-human transmission leading to sampled infections. Specifically, mutations are shown as colored circles. On the right, it shows the corresponding sequences, also with mutations shown as colored circles. We can see the sequences that share the same mutations group together. Sequences appear to be linked by a flat vertical line, which means that there are no differences between them—their sequences are identical. When a sequence sits on a long line on its own, this means that it has unique mutations not found in other sequences. The longer the line, the more mutations.

## 4. Application of Big Data Analytics in the Prevention and Control of COVID-19

After the outbreak of COVID-19 in December 2019, it quickly spread across the world [9]. On 30 January 2020, the World Health Organization (WHO) officially declared the epidemic a public health emergency of international concern. As of 3 March, it had spread to 74 countries around the world [9]. Chinese national government has carried out a rapid response, unified deployment, and strict prevention and control, taken measures such as home isolation and adjustment of the time for resumption of work and school strictly, and so on, to reduce the movement of population. By 24 February 2020, the epidemic situation in China was widely under control, and orderly business resumption had begun. However, the spreading of COVID-19 had been inexorable, and the WHO declared COVID-19 a global pandemic, including more countries on 11 March 2020. Although the fight against COVID-19 has lasted more than eight months globally, the situation remains serious and unpredictable.

**Figure 6:**
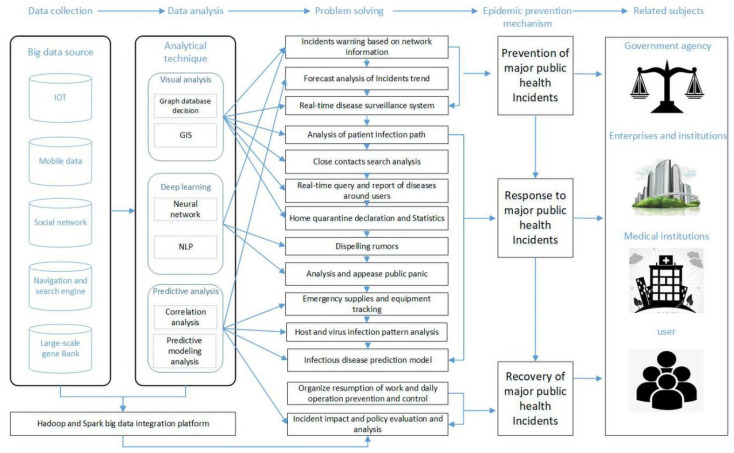
Decision framework for fight against major public health incidents based on big data analytics.

In retrospect, in the early stages of the outbreak and spread of COVID-19, a comprehensive set of scientific data analysis methods were necessary to make effective prevention decisions. As mentioned above, with the popularization of Internet technology, people can easily communicate, interact, shop, learn, and so on. Therefore, with the help of appropriate technology to analyze the large amount of data accumulated in the information network, we can effectively make accurate predictions on the spread of COVID-19, the mode of spread, the next stage of spread, and so on, to formulate corresponding policy measures. The prevention and control of major public health incidents are mainly classified into the stages of epidemic situation monitoring, early warning, disaster prevention, disaster relief, and recovery [40]. Based on our discussion of the above data sources and technologies, we will focus on establishing and improving three epidemic prevention and control mechanisms via the application of big data, as shown in Figure 6: prevention, response, and recovery.

### 4.1. Visualization of Epidemic Prevention and Control

Using visual analysis technology, it is possible to uncover relationships within massive datasets and enable investigators to obtain more intuitive visual cognition and efficient support for decision-making. At present, government and relevant policy-makers may use the above-mentioned big data sources to conduct a visual analysis of epidemic situation monitoring, medical resources, hospital enterprises, and close contact screening to make decisions. Visualization is mainly achieved through Geographical Information Systems (GIS). GIS is composed of computer hardware, software, and different methods. It is used to collect, manage, process, analyze, model, and display spatial data to solve complex management and planning problems [41]. By linking big data, GIS can assist individuals and organizations to gain a better understanding of their spatial patterns and relationships. GIS technology should first be supplied with the appropriately acquired big data. Such data collection is no longer limited to traditional equipment and methods such as total stations, satellite remote sensing, and field measurement, but it can also come from the multiple data sources discussed above. In terms of processing and analyzing, this tends to occur mainly through batch processing technology such as MapReduce and distributed system infrastructure such as Hadoop. The data source can be transformed into a digital format that is acceptable to a GIS. Based on the control of spatial data errors, the vector data topological relationship is automatically established to realize the spatial data coordinate transformation and compression processing, as well as spatial data query and analysis [42].

**Figure 7:**
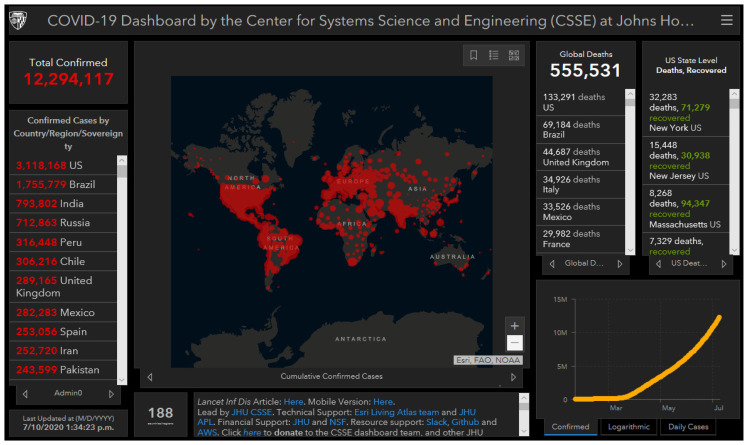
Visualization of the global development of the COVID-19 epidemic [45].

At present, governments of all countries use visual analyses of big data for the real-time visualization of key COVID indicators such as case data, epidemic distribution, epidemic situation trends, and hot spot reports. The technology can satisfy the public’s right to know to the greatest extent, and it is convenient for policy-makers to grasp the epidemic situation as a whole and support scientific decision-making. For example, Google Maps uses interactive digital maps and a virtual earth for the real-time visualization of epidemiological data. Its digital platform can also be integrated with other projects (e.g., animal and public safety) for the continuous development of the platform. For example, in 1993, the Health Mapping and GIS project jointly established by the World Health Organization and the World Children’s Fund established a global real-time monitoring network for health outbreaks, HealthMap [43]. This allows an intelligent integration of information sources for multiple disease outbreaks. These responsive, high-volume surveillance systems scan various structured and unstructured online reports to identify and track new disease outbreaks and other health problems. There is also a mobile application, Outbreaks Near Me, that cooperates with HealthMap to provide real-time monitoring of public health safety [1,44]. Figure 7 provides an example of an online GIS supplied by Johns Hopkins University, with COVID-19 data from 10 July 2020.

In addition to traditional GIS, citizens may adopt voluntary geographic information (VGI) or crowdsourcing mapping to use information provided by user resources to form an implementation map for epidemic transmission, such as OpenStreetMap [46] and Geowiki [47]. In the fight against the Ebola virus in West Africa in 2014, utilizing the VGI method, online volunteers applied satellite images to map three cities in Guinea, including 100,000 buildings, to monitor the epidemic. In addition to the Global HealthMap, during the epidemic control process of the COVID-19 virus in China, Tencent, Baidu, Lilac Garden, and others launched epidemic tracking visualization. For example, Baidu’s real-time map of the epidemic and the Baidu map migration big data platform [9] launched on 22 January 2020 enable epidemic monitoring, spring migration and epidemic trend visualization, which play an important role in supporting the further spread of the epidemic. On this basis, methods such as crowdsourcing cartography can be further combined to concentrate public resources for implementation monitoring.

Hospital resource monitoring can also be carried out through the visualization of existing data, such as the number of hospitals, medical staff resources, hospital bed and equipment resources, inpatients and other information, including real-time update monitoring and analysis. This can help strengthen the management of hospital resources and improve public perception. In order to ensure the supply of medical materials, the operation and management of medical material supply enterprises can also be scrutinized through visualization to provide support for the management and deployment of large-scale medical resources. During the resumption of economic activity in the economy (e.g., production), the real-time monitoring of an enterprise’s epidemic prevention situation can also be performed through visualization to ensure a smooth continuation of operations.

### 4.2. Screening of Close Contacts

One problem in COVID-19 epidemic prevention and control is to find potential close contacts carrying the virus. According to the epidemic transmission matrix (using the example of China), the population can be divided into four types: (1) those having a history of contact with infected cases or traveling to high-risk area (A); (2) strangers who may be in contact with A (B); (3) friends or relatives who are in contact with A (C); and people who can be separated at home (D). If you find A, you can directly find B. The most difficult problem is to find out the potential contact group B, and other people who may have been infected by B. One of the methods that can be used is to isolate potential contacts through the isolation period. In addition, through the use of a big data graph database, we may use the graph database to search for close contacts to clarify the infection process of cases and the path of disease transmission. Taking a person as a node, the spreading process of the new coronavirus is a dendritic process from one node to the contacted node. A huge network structure is formed between different nodes. At this time, a graph database can be used to store data such as related personnel, geographic location, infection time, and so on, to establish a graph model so as to realize the propagation path visualization. The establishment of a national big data system and the popularization of mobile devices provide the premise for analyzing massive behavioral data. Compared with traditional on-site reporting and statistics, this new paradigm can reduce manpower and material resources and lower the risk of personal concealment. It can also identify and locate suspected patients efficiently and inform contacts to isolate themselves. This method can assist the work of medical and community workers and reduce their risk of infection, reduce the overall isolation time of society as a whole, and promote the recovery of economic activities [48].

In addition to the above applications, related organizations can use mobile device data to perform correlation analysis for finding potential contacts. After the spread of COVID-19 is reported, the relevant data will rapidly expand over time, researchers can integrate various data, including case reports and flight lists, with information mining through data association analysis. Related transactions or relational data may be examined to find frequent patterns, interrelationships, correlations, and the causality of attributes. For example, among these data, the use of correlation analysis for epidemic transmission chains, and the discovery of infection clues, we may discover effective information for those people with close contact quickly and take response measures as soon as possible. Using the initial booking information, IP address, subsequent train trips, hotel information, mobile operator data, and the data from association analysis, we may be able to determine relationships between bookings and confirmed or suspected COVID carriers. For example, by combining different databases related to patient data from national health sources (such as the National Health Commission in China), passenger transportation, and public security systems, along with related data resources, it becomes possible to search for close contacts swiftly. For example, the public version of the “close contact measurement instrument” and the “green code” in Alipay, developed for companies responding to the epidemic situation, can confirm whether individuals are close contacts by entering their identity information [49].

In summary, governments at all levels in countries throughout the world may conduct various searches, prevention, and control of personnel from an epidemic area (within the jurisdiction of the area) that may encounter COVID-19. This includes information on the operational trajectory, inflow, and outflow of various key personnel, so as to respond to emergencies in real time, and help prevent the further spread of the epidemic.

### 4.3. Online Public Opinion Monitoring

By applying deep learning for Natural Language Processing (NLP), a government may perform more accurate speech recognition. Such recognition can be entity recognition, automatic text classification on sensitive information, documents, reports, news, and so on. This information can be collected from the Internet and social networking platforms for the monitoring of public opinions on the Internet, early warning systems, information communication mechanisms, rumor-mining, public sentiment analysis, and public appeasement. In the application of various natural language processing technologies, automatic text classification refers to the determination of textual content categories after a set of marked categories is offered. Automatic text classification is a key step in screening data related to infectious disease epidemics [50]. Sentiment analysis or opinion mining is the analysis of text data by computer to ascertain individuals’ opinions, emotions, evaluations, and attitudes about entities such as products, services, organizations, individuals, problems, and events [51]. Key methods include Word2Vec’s word vector construction method, word vector representation, window-based neural networks, recurrent neural networks, long-period memory models, recurrent neural networks, convolutional neural networks, and some models involving memory components [52]. For example, the Word2Vec model can input a word to predict the context (skip-gram model), or conversely use the context of a word as an input to predict the word itself (Continuous Bag-of-Words model, CBOW) [53].

In the process of epidemic prevention and control, natural language processing (NLP) technology can play an active role in early warning, rumor dissemination, tracking disease dynamics, social hot spots, and information push. In terms of early warning, researchers have used word embedding to classify unstructured text data, such as for example in research in the context of outbreaks of 2014 Ebola and 2016 Zika virus. For a smaller domain-specific input corpus, the Twitter corpus is better than general pre-training methods such as Word2Vec (from Google News) or GloVe (from the Stanford NLP group) in terms of extracting meaningful semantic relationships, and skip-gram’s accuracy is better than that of CBOW [54]. Using this method, we can unearth the relevant factors that will affect the epidemic, even including abnormal weather changes [55], economic development [56], and other information, for a more comprehensive early warning. The BlueDot automatic epidemic surveillance system established by Kamran Khan monitors outbreaks of infectious diseases by collecting more than 100,000 articles every day [57].

Deep learning and NLP can also be used to refute rumors during an outbreak [58]. Facebook recently launched a rumor review mechanism in response to excessive pressure and accusations of rumors. Toutiao, the Chinese news and information content platform, refute rumors based on 600 million users of big data and personalized recommendations. It can immediately stop the spread of rumors and make accurate clarification for those who have previously seen the rumors [59]. In addition, the use of deep learning to track disease dynamic text information can play a role in disease warning. Taking www.healthmap.org as an example, the website continuously tracks the content of global public health events mentioned on various online platforms.

Overall, these big data text analytics approaches can effectively help governments and other organizations grasp current public opinion in real time. Such knowledge equips decision-makers with improved monitoring intensity and response efficiency within the network of public opinion in order to implement necessary actions to manage the situation.

### 4.4. Analyzing the Virus Host Using Deep Learning

Finding the natural host, intermediate host, and final host of a virus is an important measure to prevent the outbreak of a virus. In order to clarify the host and pathogenicity of a new virus, scientists can now use a deep learning model to perform a wide-area search of virus genetic data. The partial similarity between the DNA sequence of the new virus and the DNA sequence of the known virus gene bank makes fuzzy predictions of the new virus host. Deep learning is a new research direction for machine learning; it is a supervised pattern recognition analysis method (e.g., image recognition and natural language recognition) that can recognize text, images, and sound data through the learning of the inherent laws and expression levels of sample data. Typical deep learning models include convolutional neural network (CNN), deep belief network (DBN), and stacked auto-encoder network (SAN) [60]. In terms of epidemic prevention and control, convolutional neural network model technology is the most widely used. Based on these statistical models, researchers can use deep learning to find virus hosts faster than ever during an outbreak. Zhu et al. [61] used a two-way convolutional neural network to find the host of the new coronavirus and construct a Virus Host Prediction (VHP) model, where each virus sequence is represented by a thermal matrix of its bases and codons. The “two-way” convolutional neural network implies that the same dataset will be extracted for the same structure of the convolutional neural network input. In order to use this difference to mine the deep features of the image, an image classification algorithm for a two-way convolutional neural network model is proposed. The study speculates that the bat coronavirus has a more similar infection pattern of the new coronavirus as compared to coronaviruses that infect other vertebrates. The six genomes of the new coronavirus are all very likely to infect humans.

### 4.5. Epidemic Analysis and Prediction: Infectious Disease Dynamic Transmission Model and Big Data

Big data can provide training materials for epidemic spread models, which can be used for model iteration to obtain more optimized model parameters and thus improve the accuracy of model prediction. In epidemic prevention and control, analysis with big data can predict the spread of disease and its future impact. Grover and Aujla [62] proposed machine learning techniques and algorithms to train a model and analyze an ongoing epidemic via Twitter data, using the Markov chain model to divide epidemic activity into three stages (epidemic initiation, spread, and regression) and develop a new epidemiological prediction model. Various mathematical models have also been applied to social media data for analysis, including simple susceptible–infectious–susceptible (SIS) and susceptible–infectious–recovered (SIR) models [63], and complex host–vector propagation models [64,65]. The dynamics of infectious diseases are based on the characteristics of population growth, the occurrence of diseases, and the laws of spread and development in the population, as well as the social factors related to them, which are typically used to establish a mathematical model. Through the qualitative analysis, quantitative analysis, and numerical simulation of a model’s dynamic behavior, it is possible to analyze the development process of a disease, reveal the epidemic law, predict the change trend, and analyze the causes and key points of the disease epidemic.

SIR (susceptible–infected–recovered) is the more commonly used warehouse model proposed by Kermack and McKendrick [66] when studying the popularity of Black Death in London. In 1932, the SIS (susceptible–infected–susceptible) model was established. Based on research using these models, the threshold theory in the dynamics of infectious diseases was proposed. The SEIR model of virus latency divides the population within the epidemic of infectious diseases into four categories: susceptible, exposed, infectious, and removed (SEIR). Due to differences in infectious diseases, different models tend to consider different groups of people. For example, the SEIR model is suitable when considering an incubation period for infectious diseases. Healthy people who come into contact with carriers do not get sick immediately but become pathogens. The usual assumption is that the population remains constant, the patient has a certain degree of contagion once in contact with the susceptible, and the number of people removed from the infected person per unit of time is proportional to the number of patients. Based on this, it is possible to establish a system of differential equations for various groups of people over time, which in turn can predict the inflection point and peak value of the epidemic [67,68]. For example, Dorigatti et al. [68] adopted a parametric model and a non-parametric model to estimate the lethality of the new coronavirus.

The combination of these infectious disease models along with the real-time monitoring of big data and spring rework data can more accurately predict the trend of an outbreak. Wu et al. [69] considered the impact of the quarantine policy in China and inferred the spread of the new coronavirus domestically and internationally. They combined officially provided monthly flight booking data from aviation guides, the flow of people from more than 300 prefecture-level cities in mainland China from the Tencent database, and confirmed case data from the China National Health Commission. A susceptibility–exposure–infection–recovery population model was established to simulate the epidemic in all major cities in China. The Markov chain Monte Carlo method was also used to estimate the number of basic reproductions, and the predicted scale was expressed using the obtained posterior mean and a 95% confidence interval.

Under the strict measures of joint defense and group control implemented by the Chinese government and the hard work of the medical staff, the public’s awareness of self-protection continued to increase, and the epidemic situation was gradually controlled in China. Based on the SEIR model, Yang et al. [70] and other researchers from the Zhong Nanshan team considered the role of the Chinese government’s prevention and control policies and used artificial intelligence methods to train the SARS data in 2003 to predict the development trend of COVID-19. They predicted that it would appear in middle and late February and then gradually decline until the end of April in China. The researchers predicted the development of the epidemic situation in 34 provinces in China based on the artificial intelligence deep auto-encoders (DAE) method, whereby it was concluded that the epidemic would end in mid-April. Due to uncertainty regarding the influencing factors for the epidemic, such as the second peak, the prediction will not be completely accurate, but under the relevant assumptions (such as a second peak), the development trend of an epidemic can be predicted. This form of modeling and research serves as an important reference for policy-makers contemplating the resumption of work, economic recovery, and social repair.

## 5. Challenges and Suggestions Regarding Big Data Analytics and COVID-19 Control

### 5.1. Data Reliability

The trustworthiness of big data has always been highly controversial due to data completeness, accuracy, credibility, consistency, and validity problems. The big data sources for the control of COVID-19 also suffer from these limitations and rarely capture the complexity of all social dynamics everywhere. Huge amounts of information are generated online with the continuous spreading of the disease, but the value of them is hidden behind the noise in the data. Therefore, the preprocessing of the raw data, including data cleaning, transformation, and the reduction of missing and noisy data, is necessary [19]. Before investing in this work, organizations should also identify the areas where big data could provide value for controlling the disease, estimate the impact of poor-quality information, confirm the data’s current reliability, and determine how much investment might be appropriate. Responsible and authoritative institutions may rather provide cleaned and reliable data directly. For example, the public Github repository maintained by the Johns Hopkins University Center for Systems Science and Engineering has developed to become a standard resource for individuals interested in analyzing the spread of COVID-19. The codes for tidying these data are also developed and shared on a website [71]. In pandemic and post-pandemic periods, the constant updating and tweaking of information is also necessary for reliable prediction and simulation.

### 5.2. Security and Privacy

Big data is a double-edged sword. Although big data plays an active role in confirming potentially infected cases, it also causes increasing concern among the public about security and privacy problems. There is a trade-off between the public’s right to know and privacy regarding infected cases. People are also reluctant to access similar platforms that show their locations or other related information, which becomes a barrier to the better application of big data for disease prevention. To solve this contradiction, regulation and legislation for privacy protection are impending. For example, the ICO (Information Commissioner’s Office) in the UK, EDPB (European Data Protection Board) in the EU, Israel, and South Korea have all published statements regarding personal data use during the COVID-19 crisis or amended legislation for using mobile data in tracking infected cases [72]. Obeying these regulations, a balance between the technology application and personal privacy protection can be kept by considering the following principles: collecting an appropriate amount of data, data analysis of unbiased groups, ceasing collection of data in unnecessary situations, transparency of data use, and following formal procedures.

### 5.3. Collaboration

As an unprecedented pandemic, the COVID-19 outbreak is a global crisis: a public health emergency of international concern that threatens the lives and well-being of the world population. As the COVID-19 outbreak is a global problem, it also requires a global solution. Therefore, global collaboration is vital, i.e., working together on COVID-19-related big data sources and applications (policies, best practices, and research). However, the open flow of information barely exists in healthcare. Easy collection and shared access to reliable global medical data and insights are still lacking; for example, there is an absence of epidemiological and clinical data [73]. Notwithstanding, there is also a notable trend in the deceleration of globalization and an increase in protectionism [74], which raises barriers to collaboration in big data analytics for disease prevention. Therefore, we suggest the facilitation of international collaboration to improve responses to the pandemic, following the reverse flow of the framework shown in Figure 6. The collaboration can be initiated by related agents in the world, including government agencies, enterprises, institutions, medical institutions, and individuals. For example, the WHO and many scientific research institutes play a leading role in such collaboration. It is worth noting that the scope of such collaboration for big data and COVID-19 should be encouraged to be multidisciplinary and across a spectrum of countries with different levels of economic development, including less developed countries [4]. Thus, a global collaboration mechanism for epidemic prevention can be negotiated and established for problem solving, big data analysis, and data collection. Related projects can be developed and funded to facilitate new technologies using big data for curbing COVID-19.

### 5.4. Miscalculations of Big Data

One limitation and challenge of big data and related information collection is that the misrepresentation of complex social realities can result in dangerous consequences for public health and human rights. This means that while technology may be good at collecting and analyzing digital data, a lack of access to the information behind these trends can lead to bad decisions. For example, the 2014 to 2016 Ebola epidemic in West Africa demonstrated miscalculation using big data, with inappropriate assumptions on people’s movements from call records [75]. Therefore, big data applications for preventing diseases must be judicious and employ careful research design and technologies from professionals with experience. In addition, the combination of various data sources and technologies will provide further support. More qualitative observations of COVID-19-related social factors, on top of the big data collected, are also keys to finding the truth hidden in the big data.

## 6. Conclusions

This study focuses on the application of big data analytics technology in the prevention and control of major public health incidents. First of all, we clarified the definition, characteristics, and prevention difficulties of major public health incidents. To cope with these difficulties, the use of big data is an important means to assist prevention and control in major public health incidents. Governments may make full use of the application of big data in an epidemic situation in all aspects of prevention and control, and they can further improve the epidemic prevention mechanism based on big data analytics. In terms of information collection, data collection platforms for the Internet of Things, mobile devices, navigation and search engines, social media, and large-scale gene banks can be fully established. On the basis of information collection, it is necessary to establish an early warning detection mechanism for big data analytics, e.g., using visual analysis, deep learning, and forecast line analysis techniques. This may be used as a basis for early warning and forecasting, formulating plans, rapid decision-making, and starting emergency mechanisms.

Second, governments can further improve their epidemic response mechanisms based on big data analytics. Big data technology can be used for disaster identification, decision support, coordination and communication, and technical support. Disaster identification typically uses predictive analysis of infectious disease dynamic models combined with data to make predictions regarding the criticality of an event, provide support for management decisions, report, and take timely response measures. Graph database analysis and geographic information systems can provide a significant advantage in tracking infected persons and their contacts, thus determining the source of infection. For research into virus sources and the research and development of specific drugs and vaccines, we should fully utilize the potential of big data technology for research support and technical consultation regarding genetic data and real-time patient data transmitted by the Internet of Things.

Third, the government should establish an epidemic repair mechanism based on big data analysis and promoting the sharing of big data in different regions, industries, and platforms. This includes the use of big data to eliminate fear, for recovery, audit assessment, and policy adjustment. Big data analytics can be convenient in ameliorating public fear by revealing the real-time epidemic situation and clarifying rumors. The big data analysis model can also be used to estimate the impact of the epidemic on political, economic, and social development, so as to assist governments to make suitable decisions, make policy adjustments, integrate prevention and resistance measures, and promote a rapid economic recovery.

It is important that big data analytics is merely a supportive method to assist in ex-ante prediction and ex-post prevention and control. Big data analytics has certain limitations and application premises. For instance, in terms of predicting in advance, some methods of early warning with big data may be directly used for diseases that scientists already understand, such as influenza, because relevant massive data have been accumulated. However, faced with the first generation of new viruses, it may not be possible to directly generate closely related big data, so it may not be possible to use big data technology for immediate analysis. Notwithstanding, researchers may explore the combination of data from other relevant sources for analysis, such as the incidence of internal and external causes (climates, other epidemics, etc.) that could affect how a virus is produced, and the probability of the occurrence of new viruses being observed and analyzed in advance. Moreover, the application of big data in epidemic prevention and control must consider the practicability of administrative rights, privacy protection, cost, and so on, and the balance of interests with public epidemic prevention so as to ensure operability.

## Figures and Tables

**Table 1:** Characteristics of COVID-19 incidents. WHO: World Health Organization.

Feature	Explanation	COVID-19
Sudden	The incident can suddenly erupt without warning.	Sudden outbreak.
Uncertainty	Knowledge of viruses may be limited.	Unknown, new coronavirus.
Unpredictability	The impact and sustainability of the event cannot be predicted quickly and accurately.	Political, economic, social, cultural, and other influences.
Highly hazardous	Damage to people’s health and property.	More than 21 million cases have been diagnosed worldwide, and more than 7,700,000 deaths [6].
High social attention	Arouse widespread and in-depth public attention.	Baidu index daily average of 35,083 [7]. The Google trends index reached 100 (represents the hottest search) [8].
Chain reaction	The incident occurred beyond its administrative area, expanding the scope of its impact.	Spread quickly to 188 countries in the world [9].
Timely disposability	Governments respond quickly with strict controls.	A WHO Global Emergency [6].
Preventive actions	Minimize the pandemic loss, so that the pandemic is gradually controlled; resume work and production on the basis of establishing strict avoidance and prevention experience.	Strictly control the source of infection; cut off the route of infection; protect susceptible people; introduce quarantine and curfew.
